# Mapping the 3D genome architecture

**DOI:** 10.1016/j.csbj.2024.12.018

**Published:** 2024-12-23

**Authors:** Ghazaleh Tavallaee, Elias Orouji

**Affiliations:** Princess Margaret Cancer Centre, University Health Network, Toronto, Ontario, Canada

**Keywords:** 3D genome architecture, Chromatin conformation capture, Hi-C, Single-cell genomics, Epigenomics, Chromatin

## Abstract

The spatial organization of the genome plays a critical role in regulating gene expression, cellular differentiation, and genome stability. This review provides an in-depth examination of the methodologies, computational tools, and frameworks developed to map the three-dimensional (3D) architecture of the genome, focusing on both ligation-based and ligation-free techniques. We also explore the limitations of these methods, including biases introduced by restriction enzyme digestion and ligation inefficiencies, and compare them to more recent ligation-free approaches such as Genome Architecture Mapping (GAM) and Split-Pool Recognition of Interactions by Tag Extension (SPRITE). These techniques offer unique insights into higher-order chromatin structures by bypassing ligation steps, thus enabling the capture of complex multi-way interactions that are often challenging to resolve with traditional methods. Furthermore, we discuss the integration of chromatin interaction data with other genomic layers through multimodal approaches, including recent advances in single-cell technologies like sci-HiC and scSPRITE, which help unravel the heterogeneity of chromatin architecture in development and disease.

## Graphical Abstract

**Figure:**
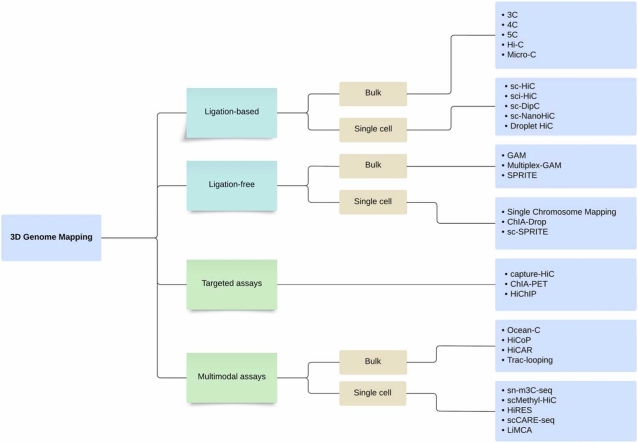

## 1 Introduction

Genome can be explored at different levels using various methodologies, depending on the aspect of interest. Until recently, methods of DNA mapping were mostly based on the linear exploration of the genome. However, the genome is a dynamic three-dimensional (3D) structure that changes based on the various cell conditions [1]. The DNA of the diploid cell is about two meter long, which is folded and packed into nucleosomes, and further coiled into “30-nanometer fiber” and higher order structures to fit in the nucleus. Higher order inter-chromosomal spatial arrangements shape the 3D organization of DNA, which is maintained by proteins and RNAs [2], [3]. Proteins that integrate into chromatin regions facilitate bringing two or more linearly distant loci to close proximity, affecting the expression of the genes [4]. The spatially close loci on chromosomes tend to interact predominantly and occupy specific regions within the interphase nucleus, forming chromosome territories (∼100 Mb). At smaller scales (1–100 Mb), they partition into two distinct hubs of active (A compartment) and inactive (B compartment) chromatin, which further fold into topologically associating domains (TADs; 40Kb-3Mb). TADs consist of early and late replication domains, and DNA loops ranging from less than 1Kb to a few Mb [5].

The genome organization has a profound impact on the DNA function. Although gene expression is affected by several factors including relative positioning to spatial nuclear landmarks, chromosome folding is a critical determinant. Chromosome folding mediates physical contacts between cis regulatory elements (CREs) and target genes, which predominantly occurs within the TADs. TADs usually have highly conserved positions in different cell types and are similar in size to replication domains [6]. Thus, modifications that happen in the composition and structure of the genes during development, biological processes or diseases can affect the 3D arrangement of the genes, which subsequently influences CRE–gene contacts and alteration of nuclear sub-compartments.

Higher order chromosomal structures have long been discovered and identified by electron microscopy and FISH, however, chromosomal conformation capture (3 C)-based technologies have advanced their visualization and dynamics at large scales. During the past two decades several techniques have been developed to investigate the 3D conformation of chromatins. These methods have helped mapping the interactions among chromosomal regions and understanding how changes in genome architecture can be associated to different cell states. Despite the increasing load of work and discoveries that have verified the importance and various roles of genome organization in the biology of the cells, there is still a lot to be unveiled in the field which calls for more advanced multimodal technologies. Thus, it is essential to learn about the techniques and tools used to explore and analyze the 3D organization of the genome and how they are further developed and advanced up to date.

The 3D genome can be interrogated using different methodologies, forming two main categories: ligation-based techniques or chromosomal conformation capture (3 C)-based methods, and ligation-free methods. Here, we review different techniques of mapping chromatin architecture, with a particular focus on Hi-C, as the majority of the 3D genome approaches leverage from this method.

## 2 Methods for probing 3D genome architecture

### 2.1 Ligation-based methods

#### 2.1.1 Chromosome Conformation Capture (3C)-based methods

**Fig. 1:**
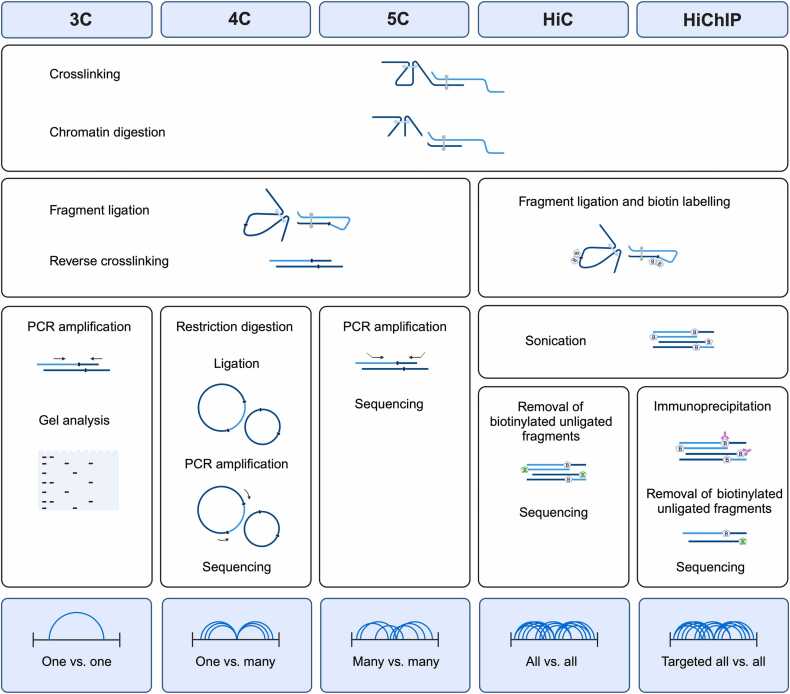
Overview of ligation-based 3D genome mapping methods. This figure provides a schematic comparison of Chromosome Conformation Capture (3 C)-based techniques. The 3Cmethod measures pairwise chromatin interactions by ligating digested chromatin fragments, followed by targeted PCR amplification. 4 C builds upon 3 C to profile interactions of a single locus with the entire genome, enabling a "one-vs-all" interaction view through circularization and inverse PCR. 5 C expands this further to "many-vs-many" interactions using multiplexed ligation-mediated amplification and sequencing. Hi-C introduces biotinylated fragment labeling to produce genome-wide, unbiased "all-vs-all" interaction maps. Finally, HiChIP integrates Hi-C with chromatin immunoprecipitation (ChIP) to enrich for chromatin interactions involving specific proteins, providing a protein-centric perspective on chromatin organization.

Considering the direct effects of the 3D genome organization on major biological processes, such as transcriptional regulation and DNA replication, focus of genomic research over the past two decades has been on developing assays to capture and characterize in situ 3D chromosome contacts and associated factors. The first of the kind, chromosome conformation capture (3 C), was introduced in 2002 by Dekker et al. [7]. Later, chromosome conformation capture-on-chip (4 C) [8], chromosome conformation capture carbon copy (5 C) [9], and high-throughput chromosome conformation capture (Hi-C) were developed (Fig. 1) [10]. Hi-C has enabled mapping all the possible loci interactions within the whole genome. Hi-C is the most popular and commonly used 3 C technique and the basis for majority of the ligation-based methodologies. Ligation-based methods have revealed the significance of chromatin structure and 3D organization on gene expression, function of regulatory elements and other features that directly or indirectly impact gene regulation. It was in the light of 3 C studies that conserved structural organization of chromosomes and regulatory principles of chromatin looping were recognized [11]. Some of the applications of Hi-C data include but not limited to reconstruction of whole genome contacts, scaffolding and assembly, haplotype and centromere annotations [12]. In the next section, we will focus on Hi-C procedure and then discuss the processing tools, and analysis pipelines.

##### 2.1.1.1 Hi-C -experimental workflow

Standard (in-situ) Hi-C assays usually consist of the following steps: (1) Fixing cells: cells are crosslinked with formaldehyde which introduces bonds that immobilize interactions between genomic loci. (2) Digestion of the DNA with restriction enzymes: chromatin is then digested with a type 2 restriction endonuclease, leaving sticky ends. (3) Filling in the sticky ends and biotinylation: restriction digestions cut the DNA at specific sequences. This cleavage site provides a template for labeling the restriction fragments with biotin ^14^dCTP. (4) Ligation of the DNA fragments: crosslinked fragments within the same chromatin complex ligate intra-molecularly. (5) DNA shearing and pulldown: shearing the ligated DNA and pulling down the biotinylated fragments. (6) Library construction and paired-end sequencing of the selected DNA fragments [10]. With these technical steps, Hi-C enables capturing of all possible proximity ligation among the genomic loci and provides a scaffold for measuring 3D distances between interacting regions in the genome.

##### 2.1.1.2 Hi-C analysis

Obtaining high quality data is one of the major challenges of Hi-C experiment. Hi-C captures the whole genome contacts, creating a very large dataset of all possible interactions, about 10^12^ possible pairwise interactions for human genome. Therefore, to obtain maximal effective resolution, the interactions are reduced to fixed-size genomic intervals to aggregate data and increase the signal to noise ratio. This process is called binning [12]. Hi-C data is usually binned into a range of 1Kb to 1 Mb sized bins depending on the depth of sequencing [13].

As binning directly affects the results of downstream analysis, a single Hi-C dataset can be binned into multiple bin sizes depending on the analysis goals. While low-resolution Hi-C datasets are usually used for large-scale genomic structures like genomic compartmentalization (A/B) or TAD boundaries, to define sub-domains (subTADs) or enhancer–promoter interactions (loops), high-resolution datasets are needed [14].

When Hi-C data is properly binned, interaction frequency (IF) can be reliably determined. Interaction or contact frequency refers to the number of interactions between a pair of chromosomal or genomic regions in the Hi-C data, that can happen between fragments (bins) within a chromosome (intra-chromosome) or between different chromosomes (inter-chromosome). IFs for all pairwise regions or loci are combined to represent a symmetric matrix called IF matrix, contact matrix or contact map. Thus, they play fundamental roles in defining 3D structure and features of the DNA including genome folding, gene regulations, and the connection between regulatory elements. The rows and columns of the matrix correspond to the equal size of the bins referred to as the Hi-C resolution [13].

Major determinants of resolution for a Hi-C library are depth of sequencing, choice of restriction enzyme and library complexity [15]. There are multiple restriction enzymes that can be used individually or in combination to generate Hi-C libraries which include restriction enzymes that recognize sequences of 4–8 bps. The *Hin*dIII generates DNA fragments of ∼4Kb, whereas *Mbo*I and *Dpn*II are predicted to produce 7–9 times smaller fragments, creating higher resolution Hi-C libraries. Library complexity is another defining element of Hi-C resolution that is described as the total number of unique chimeric construct in a Hi-C library. Libraries with lower level of complexity are less preferred, as they saturate quickly with increasing read depth [12].

##### 2.1.1.3 Mapping

Hi-C reads are usually aligned to the corresponding genome by standard read alignment softwares such as Bowtie2, BWA and BWA-MEM2 [16], [17], [18] The output of aligned reads is usually in the format of a sequence alignment and map (SAM) or binary alignment and map (BAM) files. A range of tools and pipelines have been developed for Hi-C data analysis, including but not limited to HiC-Pro, HiCUP, HiCExplorer, Juicer, HiC-TE, FAN-C, and SnakePipe [19], [20], [21], [22], [23], [24], [25], [26]. Each framework offers unique features: HiC-TE specializes in analyzing interactions involving transposable elements (TEs) [25], FAN-C provides a multi-featured command-line interface with flexibility for various input data formats [21], and SnakePipe and HiCExplorer enable the integration of multiple assay types into cohesive workflows [19], [23].

##### 2.1.1.4 Filtering

Following mapping of the reads to the reference genome, valid reads need to be determined by applying standard filters, and the uniquely mapped reads are selected for further processing. The next step is assigning each mapped read to the nearest restriction site based on the restriction enzyme and the reference genome [12], [27], [28]. Once each of the paired-end reads is assigned to a single fragment, the next step is to filter out invalid ligation products including dangling end and self-circle ligation. Additional stringent filtering of all possible artifacts such as PCR duplicates and undigested restriction sites is also performed to finally proceed with only pair-end reads that provide information about proximity ligation of genomic regions [12], [27], [28].

##### 2.1.1.5 Normalization

Hi-C datasets contain noise and biases which requires a number of normalization steps being done to be able to identify meaningful biological interactions. These correction approaches are generally divided into explicit or implicit models. While explicit models balance the known sources of bias, such as GC content, fragment length, mappability and copy number [29], [30], [31], implicit models correct for all unwanted variation in the contact map without considering sources of bias [11], [32], [33], [34].

Current advancements in technology have improved the quality of recent normalization methods. Band-wise normalization and batch correction (BNBC) has enabled comparison of the contact maps across different samples at the level of individual contact cells [35]. There might be high correlation between correction factors of different normalization approaches [11], and each technique must be chosen based on the downstream procedure and the purpose of the study [36].

##### 2.1.1.6 Visualization

Once the Hi-C data is normalized, the next step is to visualize the contact maps of the corrected reads. Contact matrices are usually represented as symmetrical square heatmaps with pixel intensity corresponding to the contact count, or circular plots in which contacts are indicated by edges that connect distal pairs of loci. Hi-C data visualization depicts spatial interaction of genomic loci, unveiling 3D chromatin features with each geometric shape representing a specific biological process. Various tools are available for Hi-C data visualization, as detailed in the next section. These tools often accept inputs generated by one or a combination of mapping pipelines such as HiC-Pro, Juicer, GENOVA, and HiCExplorer [22], [23], [37], [38].

##### 2.1.1.7 Organizational patterns in Hi-C

As mentioned briefly in the introduction, chromosomes tend to occupy certain regions in the nucleus which are called chromosomal territories. On a Hi-C map, chromosomal territories are observed as small triangles of enhanced contact frequency that tile the diagonal of the contact matrix [39]. Chromosomal territories further divide into two distinct compartments; compartment A which is transcriptionally active and B that is repressed. Compartment A is known to have high GC content and comprise euchromatic gene-dense regions while compartment B has low gene-density and makes heterochromatic regions. Gene-dense regions refer to the chromatin domains with high gene count per unit length of genomic sequence [40], [41]. A/B compartmentalization are represented as squares in the contact matrix [42]. Topologically associating domains (TADs) are functional regions in the genome where loops form between regulatory elements and genes, resulting in a high frequency of internal interactions. These domains appear as rectangular regions of elevated interaction frequency (IF) on contact maps and are defined by boundary regions where there is a sharp decline in local interactions. These boundaries, believed to correspond to insulator or barrier elements in the genome, are particularly enriched with CTCF-binding sites and cohesin complexes, which play crucial roles in maintaining the structural organization of TADs [43]. At smaller scales of sub-megabase, some chromatin domains called sub-TADs shown as corner-dot domains that are nested within larger TADs. Sub-TADs are formed mechanistically by extrusion and may or may not co-localize with compartments [44]. Enhancers can directly connect to promoters via CTCF-dependent and CTCF-independent mechanisms, forming corner dots on the contact map. When corner dots co-localize with chromatin domains at their apexes, they form chromatin loops by a mechanism called loop extrusion [44]. Given the enrichment of CTCF in TAD boundaries, TAD’s locations can be in part indicated by CTCF binding sites that arrest loop extrusion via cohesin complex. Chromatin loops manifest as points in the Hi-C map [45].

Genomic regions with high levels of local chromatin interactions are called frequently interacting regions (FIREs). Like chromatin loops, FIRE formation is also partially dependent on CTCF and the cohesin complex. FIRE regions are enriched within TADs and toward the TAD center, however, they are depleted close to TAD boundaries. FIREs are conserved between human and mouse. They are usually enriched near genes involved in tissue function and exhibit strong tissue-specificity in local chromatin interactions. FIREs are enriched for super-enhancers; they are hot spots of local chromatin interactions and most likely interact with several cis-regulatory elements in their TADs. They are believed to help annotate disease-associated non-coding single-nucleotide polymorphisms (SNPs) [46].

**Fig. 2:**
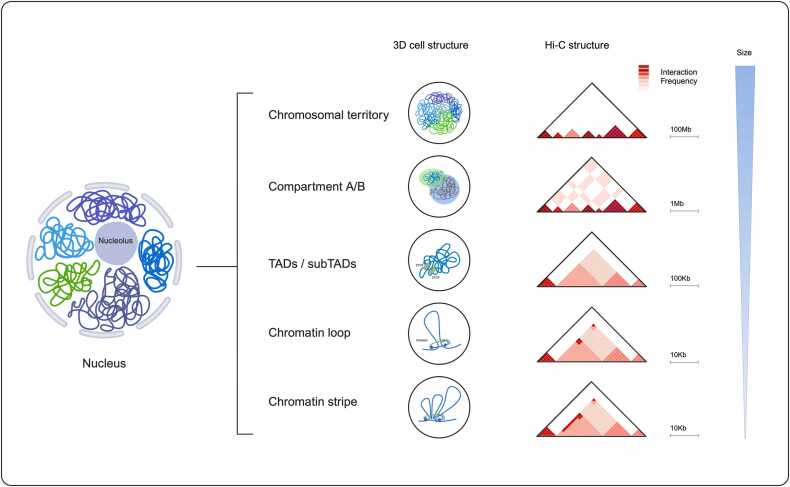
Hierarchical 3D genome structures and Hi-C representations. This figure illustrates the hierarchical organization of chromatin within the nucleus and how these structures are visualized in Hi-C maps. Chromosomal territories represent distinct spatial regions occupied by individual chromosomes, seen as large, sparse contact domains. Compartments (A/B) reflect the segregation of active and inactive chromatin regions, forming characteristic checkerboard patterns on Hi-C maps. TADs and subTADs are self-interacting genomic domains, appearing as squares along the diagonal, indicating frequent local chromatin interactions. Chromatin loops form between specific loci, such as enhancers and promoters, and are visualized as sharp peaks outside the diagonal. Chromatin stripes are elongated interaction patterns extending along the diagonal, often representing loop-anchored regions such as active transcription sites. A schematic scale highlights the size range of these features, from chromosomal territories at hundreds of megabases to chromatin loops at tens of kilobases.

Another structure in the Hi-C map that is generally associated with active enhancers is architectural stripes. Architectural stripes form where a single locus (the stripe or loop anchor) interacts with a contiguous genomic interval or so called the “stripe domain” at high frequency. They tether super-enhancers to their corresponding promoters. Stripes range from a few to hundreds of Kb and can be seen along the edges of domains with abundant cohesin loading (Fig. 2) [47], [48].

Visualization of Hi-C data can be challenging due to dimensionality and complexity of the genomic networks and interactions, however, advances in bioinformatic softwares, has evolved processing and visualizing Hi-C data towards easier and more user-friendly operating tools. It is now important for visualization tools to be able to simultaneously process Hi-C data along with other chromatin modalities, to compare and integrate various features throughout the genome [49], [50]. There are web-based tools such as HiGlass [51], HiBrowse [52], my5C [53], Juicebox [38], the WashU Epigenome Browser [54] and the 3D Genome Browser [55], readily accessible to visualize Hi-C data sets.

Hi-C visualization tools have their own advantages and limitations. HiGlass and Juicebox provide interactive Hi-C heatmaps that can be used at different resolutions and have the ability to visualize maps together with other genomic data such as ChIP-seq and synchronously compare multiple maps. The heatmap generated with WashU Epigenome Browser or the 3D Genome Browser is displayed as a triangle and rotates by 45°, useful in displaying chromatin conformation at selected loci [38], [51], [52].

Hi-C visualization tools often specialize in detecting and analyzing genomic features like compartments, differential compartments, insulation scores, TADs, loops, stripes, and differential contacts. Tools such as Juicebox, FAN-C, mustache, HiCExplorer, GENOVA, dcHiC, Selfish, FitHiC, and Strippen enable these analyses [20], [21], [37], [38], [56], [57], [58], [59], [60], [61]. HiCExplorer, for example, performs additional tasks such as TAD calling, which can be incorporated into Hi-C heatmaps [23]. dcHiC enables comparative analysis across multiple contact maps, identifying biologically significant compartmentalization differences. It supports pairwise differential analysis and multivariate differential comparisons, allowing simultaneous analysis of multiple Hi-C maps without generating numerous combinations. GENOVA, an R package, offers specialized visualization and analytical features, such as relative contact probability plots, insulation scores, compartmentalization analyses, and TAD/loop aggregation [56]. Other Hi-C visualization tools include packages such as HiCplotter [62] and HiTC [63] that can be applied to the contact maps.

snHiC, a Snakemake-based pipeline, generates contact matrices at multiple resolutions in a single run. It aggregates individual samples into user-specified groups and integrates domain, compartment, loop, and stripe detection while facilitating differential analysis between sample groups [20]. HiBrowser, another comprehensive tool, enables local deployment for visualization and analysis of Hi-C data alongside genetic and epigenetic annotations. It supports multi-sample visualization for intuitive comparisons across datasets, offering a dynamic, multi-omics navigation system [64].

HiCeekR and Genome Interaction Tools and Resources (GITAR) are also able to generate interactive plots [50], [65]. HiCeekR enables the user to select two main representations: Heatmap and Network. By combining computational and statistical methods, HiCeekR can identify genome compartments, TADs, and integration of Hi-C data with other genomic datasets, such as ChIP-seq, RNA-seq, the functional analysis, and the visualization of the interaction network [65].

#### 2.1.2 Micro-C

Given that 3C-based techniques rely on restriction digestion of the genome, the resolution of the data is affected by heterogeneity of the restriction target sites. Thus, Hsieh et al. developed a Hi-C-based method named Micro-C, for nucleosome-resolution chromosome folding studies in which (1) chromatin is fragmented into mononucleosomes using micrococcal nuclease. (2) Fragments are subjected to mononucleosomal end repair. (3) Ligation products are purified by a modified two-step protocol. (4) Paired-end deep sequencing and data analysis [66]. Micro-C uses MNase to digest cross-linked DNA in regions that are not stably bound by proteins across the genome, resulting in mono, di, or tri-nucleosome sized fragments. Thus, it can specifically be used for short-range analysis of nucleosome fiber folding, which is not detectable in Hi-C or restriction enzyme-based assays that generate multi-nucleosome-sized fragments [67]. Using Micro-C, high resolution maps for the budding yeast Saccharomyces cerevisiae were generated, depicting abundant self-associating domains which typically span one to five genes and localizing the boundary activity of highly transcribed genes to their promoters [66]. Compared to Hi-C, Micro-C was able to detect more chromatine interaction and significantly higher signal-to-noise ratios for close-range contacts in human cells [67], [68]. Micro-C captures more loops at high resolution than Hi-C, while in TAD calling there is no significant difference between the two methods [67].

### 2.2 Single cell ligation-based methods

As discussed, genome is a dynamic 3D organization with heterogeneous chromosome structures in individual cells of an organism. Although standard Hi-C has enabled mapping 3D genome contacts and structures, at bulk, the constructed contact maps represent the average of a population of cells. The emerging single-cell Hi-C (scHi-C) technologies have addressed the gap by offering unique opportunities to study the heterogeneity and dynamics of higher-order chromatin structure and function via simultaneous detection of thousands of genomic interactions in a single cell [69]. Several scHi-C methodologies have been established, each have their own advantages and significant differences in efficiency, bias, scale, and costs [70]. In this section we introduce the most common single cell Hi-C methods.

#### 2.2.1 scHi-C

In 2013, Nagano et al. established a scHi-C technique whereby they demonstrated cell-to-cell variation of the trans-chromosomal contact structures, but conservation of the domain organization in individual chromosomes. This study suggests that localization of active gene regions to boundaries of chromosome territories is a hallmark of chromosomal conformation, although there is structural stochasticity within the individual cells.

Similar to bulk Hi-C, the scHi-C procedure initiated with chromatin crosslinking, restriction enzyme (*Bgl*II or *Dpn*II) digestion and biotin fill-in. However, in the single cell method ligation is performed in the nuclei, whereas in bulk Hi-C ligation is done after nuclear lysis and dilution of chromatin complexes. Individual nuclei are selected under the microscope, placed in individual tubes, and reverse crosslinked. Biotinylated Hi-C ligation junctions are then purified on streptavidin-coated beads followed by digestion of the captured ligation products with a second restriction enzyme (*Alu*I) to fragment the DNA. Customized Illumina adapters with unique 3-bp identification tags were ligated to DNA fragments. scHi-C libraries are PCR-amplified, size selected and sequenced using multiplexed, paired-end configurations [69], [70], [71].

#### 2.2.2 Massively multiplex single cell Hi-C (sci-HiC)

Later in 2017, single-cell combinatorial indexed Hi-C (sci-Hi-C) was developed by Ramani et al. Sci-Hi-C combines Hi-C with combinatorial cellular indexing to map chromatin contacts of a large number of single cells based on the karyotypic and cell-cycle state differences of each individual cell. In this high-throughput technique, (1) cells are fixed and lysed to generate nuclei. (2) The nuclei are digested in situ with a restriction enzyme (*Dpn*II). (3) Digested nuclei are distributed into 96 wells and barcoded by ligation of barcoded biotinylated double-stranded bridge adaptors. (4) The intact nuclei are pooled, ligated, diluted, and redistributed into a second 96-well plate, with the dilution being critical to ensure that each well in the second plate carries a maximum of 25 nuclei. (5) A second round of barcoding is performed, where nuclei are ligated with additional barcoded adaptors, enabling the number of barcode combinations to exceed the number of nuclei, so that most single nuclei are tagged by a unique combination of barcodes. (6) The nuclei are pooled again to purify biotinylated junctions with streptavidin beads, followed by restriction digestion. (7) Libraries are then prepared and sequenced to generate contact maps [72], [73].

In the same year, Ke et al. used an optimized Hi-C technique with low-cell number to uncover the 3D structural changes of paternal and maternal genomes in zygotes, reporting the gradual formation of clear higher order chromosomal structures from two-cell embryos with obscure structures and domains [74].

#### 2.2.3 Single cell Dip-C

Another technique that captures chromatin conformation at single-cell level is Dip-C. This method generates phased SNPs to distinguish between the two chromosome haplotypes. Dip-C can detect more true contacts by combining a transposon-based whole-genome amplification technique named Multiplex End-tagging Amplification (META) with an imputation algorithm to assign the two chromosome haplotypes linked by each contact. In Dip-C all biotin related steps are removed from a standard Hi-C. (1) Cells are fixed, digested and ligated. (2) Ligated cells are then lysed, filtered and sorted into single cells by flow cytometry. (3) Lysates are transposed, and PCR mix is used for amplification of the whole genome. (4) Libraries are prepared by two additional PCR steps, purified and sequenced. Dip-C helped reconstructing the genome structures of single diploid human cells with high spatial resolution, locating specific SNPs in the nucleus [75].

#### 2.2.4 Single cell NanoHi-C (scNanoHi-C)

scNanoHi-C uses Nanopore long-read sequencing to profile 3D chromatin structures within individual cells. In scNanoHi-C like other proximity ligation-based methods cells are (1) fixed and crosslinked. (2) Single cells are selected by fluorescence-activated cell sorting (FACS) and deposited onto a 96-well plate. (3) 24 individual Tn5 enzyme conjugates are designed and a low-density Tn5 transposon with the same adaptor is used to randomly fragment genomic DNA from each single cell. (4) Cells tagged with different Tn5 barcodes are pooled together. (5) After primer-dimer removal, the ∼3 Kb long amplicons are sequenced on the Oxford Nanopore Technologies (ONT) platform. The sequenced reads that contain multiple ligated DNA fragments (monomers) are termed concatemers and numbered cardinally.

It is notable that up to 24 × 96 cells can be sequenced, with scNanoHi-C, in one PromethION run. This flexibility allows either detection of higher resolution genome structures within individual cells or more single cells at a lower sequencing depth. Contact matrices unveiled the extensive existence of high-order chromatin structures in active chromatin regions across the genome, and systemically identified multiway enhancer-promoter interactions within individual cells [76].

#### 2.2.5 Droplet Hi-C

Droplet Hi-C is a recently developed technique that combines the in situ Hi-C procedure with the high-throughput capabilities of the 10X Genomics microfluidic platform to profile single-cell chromatin conformation in droplets. In this technique (1) Formaldehyde-cross-linked cells are digested by restriction enzyme and ligated in situ. (2) Cells are treated with SDS to remove histone proteins. (3) DNA is fragmented and captured in a 10X-genomics microfluidic platform. (4) Cell-specific DNA barcodes are added to the DNA fragments. (5) Library is constructed and sequenced [77]. Droplet Hi-C is a relatively time and cost-efficient method that can accurately distinguish cell-type-specific chromatin organization, suggesting that the genes with higher expression variation across cell types are more associated with variable TAD boundaries. Droplet Hi-C can be used to identify abnormal chromatin structures and extrachromosomal DNA (ecDNA) in tumor cells, and map their chromatin interactome at single-cell resolution. It can also serve as a multimodal assay to simultaneously capture transcriptome and chromatin architecture in single cells [77].

Overall, single cell Hi-C techniques have enabled simultaneous discovery of numerous higher-order chromatin structures in an individual cell, confirming cell-to-cell variability of 3D genome organization that was first identified by fluorescence in situ hybridization (FISH).

### 2.3 Ligation-free methods

**Fig. 3:**
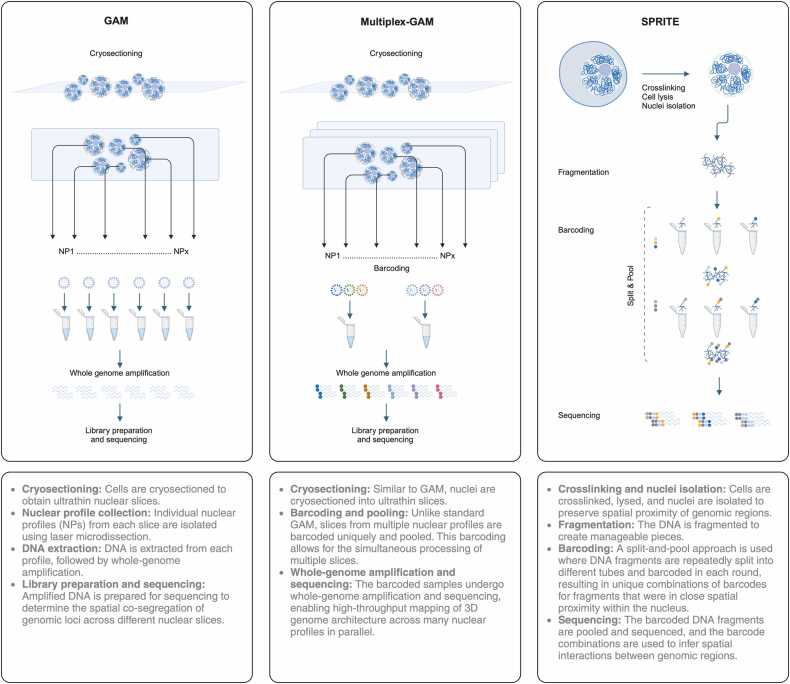
Ligation-free 3D genome mapping methods. This figure depicts the workflows of ligation-free techniques for mapping 3D genome architecture, including GAM, Multiplex-GAM, and SPRITE. In GAM, ultrathin nuclear slices are obtained through cryosectioning, followed by laser microdissection to isolate individual nuclear profiles. DNA is then extracted, amplified, and sequenced to identify loci that co-segregate across nuclear slices. Multiplex-GAM extends this approach by introducing barcoding and pooling of nuclear profiles, enabling simultaneous high-throughput analysis of multiple slices in parallel. SPRITE begins with crosslinking and isolation of nuclei, followed by DNA fragmentation. Using a split-and-pool barcoding strategy, DNA fragments are repeatedly split into different tubes and tagged with unique barcodes, preserving information about spatial proximity. Sequencing of the barcoded fragments allows the reconstruction of chromatin interactions across the genome.

In addition to 3C-based techniques, microscopy has also been widely used to study spatial genome organization. FISH can directly visualize the spatial relationship of sequences. While 3C-based techniques unveil the genomic interactions within an individual chromosome, microscopic observations suggest that genome interactions can occur beyond chromosome territories and organize around nuclear bodies [3]. The 3C-based and FISH methods are expected to show the same views of genome organization, but there are discrepancies that can affect interpretation of the data [78]. In general, reliance of ligation-based techniques on digestion and ligation poses considerable limitations which makes it difficult to quantify simultaneous contacts between multiple chromatin regions. There are also biases due to GC content, protein occupancy and restriction site density. While ligation-based methods are unable to measure chromatin associations with the nuclear periphery or chromatin compaction, microscopy approaches are also limited by the choice of probes for a known set of genomic regions [3], [78], [79]. Therefore, ligation-free techniques such as Genome Architecture Mapping (GAM) and Split-Pool Recognition of Interactions by Tag Extension (SPRITE) address these drawbacks (Fig. 3). Alternatively, the pA-DamID (protein A-DamID) technique can be used to study the dynamics of Lamina-Associated Domains (LADs) and their interactions with the nuclear lamina (LAD–NL contacts) during early interphase and DNA replication. This method utilizes an antibody-based approach to tether a DNA adenine methyltransferase (Dam) to specific nuclear lamina proteins, enabling high-resolution, high-throughput sequencing to identify LADs. Furthermore, pA-DamID allows for in situ visualization of these interactions, providing a comprehensive understanding of nuclear lamina organization [80].

#### 2.3.1 GAM

Since loci in spatial proximity in the nuclear space are usually found in the same NP, this technique allows detection of simultaneous contacts between multiple chromatin regions. At 1 Mb genomic resolution, GAM and Hi-C contact matrices show high correlation genome wide. GAM contact matrices independently confirm the existence of TADs. Principal Component Analysis (PCA) is a powerful multivariate data analysis tool, particularly used to reduce the dimensionality of datasets while maintaining essential information. GAM based PCAs and Hi-C PCAs overlap in genome compartments, but the resolution of GAM data is limited by the number of analyzed slices. In general, GAM can bypass ligation and digestion steps of Hi-C and requires lower number of cells [79].

#### 2.3.2 Multiplex-GAM

A faster and more affordable variant of GAM, termed multiplex-GAM, has recently been introduced, enabling the mapping of significantly more chromatin contacts genome-wide. In this method, (1) DNA from multiple nuclear profiles (NPs) is extracted and sequenced together, reducing sequencing costs. (2) Contact maps are generated from single-NP data as well as in silico 2NP and 3NP data through simulation of combined single nuclear profile datasets. (3) Matrices are recalculated to analyze co-segregation from the simulated multiplex-GAM datasets. (4) An improved version of the SLICE model (Statistical Learning in Chromatin Environment) is used to infer chromatin contacts by analyzing co-segregation frequencies and calculating experimental parameters. (5) Cell profiles are stained for better identification, and the data is visualized. Although GAM and Hi-C detect similar TADs and CTCF-mediated loops, multiplex-GAM appears to identify stronger and more complex contacts within active chromatin regions across longer distances [81].

#### 2.3.3 SPRITE

The Split-Pool Recognition of Interactions by Tag Extension (SPRITE) is another ligation-free method which enables genome-wide detection of higher-order interactions occurring simultaneously between multiple DNA sites within the same nucleus [3]. Experimental workflow includes the following steps: (1) DNA, RNA, and protein are dual cross-linked with disuccinimidyl glutarate (DSG) and formaldehyde in cells. (2) Cells are lysed, and the DNA is fragmented by sonication and DNase digestion. (3) Crosslinked lysate is coupled to NHS-ester beads. (4) DNA is end-repaired and dA-tailed. (5) Interacting molecules within an individual complex are barcoded using a split-pool strategy including DNA phosphate modified (DPM) ligation and reverse crosslinking. (6) Interactions are identified by sequencing and matching all reads that contain identical barcodes.

The SPRITE can generate pairwise contact maps that are highly comparable to Hi-C maps, with similar structural features observed at various genomic resolutions. In addition, as opposed to proximity ligation-based methods, measuring simultaneous interaction of multiple DNA molecules within an individual nucleus and detection of interactions with cell-to-cell variation is also possible by SPRITE. SPRITE does not need specialized equipment and nor does it require extensive amplification of the whole genome [3]. In silico data generated from Hi-C, GAM and SPRITE reveals comparable outcome for these three methods and faithful to the reference 3D structures [82].

### 2.4 Single cell ligation-free methods

With the invention of single cell assays, scHi-C techniques were established that allowed capturing chromatin conformation and dynamics at single cell level. However, confinement of these techniques by proximity ligation limitations were posing difficulties in mapping long-range and higher-order interactions. Single chromosome, and single cell ligation-free methodologies were then introduced to overcome these obstacles, which benefit from advances in microfluidics and microscopy.

#### 2.4.1 Single chromosome mapping

Spatial organization of chromatin domains and TADs were visualized in single chromosomes by Wang et al. in 2016. They invented a multiplexed FISH method to sequentially image many genomic regions for 3D tracing of individual chromosomes in the nucleus, and further implied this method to study the spatial arrangements of TADs and A/B compartments in human chromosomes 20, 21, 22 and X. In this technique, (1) numerous primary probes are hybridized to the targeted chromosome. (2) The readout sequences on the primary probes are sequentially hybridized to secondary probes targeting each TAD. (3) Nuclei are imaged and bleached. (4) The measurements are made by the aid of Hi-C maps, demonstrating spatially polarized organization of A/B compartments in single chromosomes of individual cells, as well as distinct folding and compartmentalization of inactive and active X chromosomes [83].

#### 2.4.2 ChIA-Drop

Chromatin interaction analysis via droplet-based and barcode-linked sequencing (ChIA-Drop) is another ligation-free technique for mapping multiplex chromatin interactions with single-molecule precision that barcodes chromatin complexes inside microfluidics droplets which DNA will be pooled for sequencing. ChIA-Drop takes advantage of the Chromium microfluidics system (10X Genomics) and works through following steps: (1) Chromatin is crosslinked, fragmented, and directly loaded to the microfluidics device without proximity ligation or DNA purification. (2) Chromatin complexes are individually partitioned into a Gel-bead-in-Emulsion (GEM) droplet. (3) The barcoded amplicons are pooled for sequencing to be computationally assigned to the same GEM of origin to infer multiplex chromatin interactions [84], [85].

Data generated by ChIA-Drop has revealed a high level of heterogeneity and multiplex chromatin interactions. For data analysis and visualization, the same group developed a pipeline called ChIA-DropBox. ChIA-DropBox first identifies the droplet barcode (GEMcode) from sequencing reads to map the interactions. High-quality reads with the same GEMcode are grouped together, followed by merging the reads that overlap. Interestingly, inter- and intra-chromosomal interactions can be computationally separated into sub-GEMs. This tool also filters out singleton DNA fragments, a major source of noise, in downstream analysis [84], [85]. ChIA-DropBox transforms the ChIA-Drop data into a pairwise format that can be visualized in generic 2D contact map viewers or loop visualization tools. Additionally, to visualize multiplex chromatin interactions interactively, one can use a browser specifically designed for ChIA-DropBox data [85].

#### 2.4.3 scSPRITE

Single-cell split-pool recognition of interactions by tag extension (scSPRITE) measures multiway DNA interactions and generates higher-resolution contact maps at single cell level compared to proximity ligation-based methods. In this technique: (1) Cells are dissociated into a single cell suspension. (2) DNA is crosslinked, followed by nuclei isolation and permeabilization. (3) DNA is digested by a restriction enzyme. (4) DNA fragments are subjected to two steps of split-and-pool barcoding to tag the contains of the same nucleus as well as the 3D spatial arrangement of the same fragments. scSPRITE is able to measure almost ten-fold more inter-chromosomal contacts per cell compared to scHi-C, even though the number of sequencing reads per cell is about ten-fold lower for scSPRITE relative to scHi-C. Capturing multiway DNA contacts by scSPRITE has improved the structural resolution, suggesting organization of inter-chromosomal hubs around nuclear bodies as well as heterogeneity across long-range contacts [86].

### 2.5 Targeted approaches to 3D genome mapping

Hi-C captures all possible proximity ligations in the whole genome, thus very deep sequencing is required to fully map 3D architectural landscapes of chromosome. To enhance the quality and specificity of the chromatin interactions Capture Hi-C (CHi-C) and Chromatin Interaction Analysis by Paired-End Tag Sequencing (ChIA-PET) were developed that detect locus-directed or protein factor-directed chromatin interactions respectively.

#### 2.5.1 Capture Hi-C (CHi-C)

Capture Hi-C integrates Hi-C with the hybridization-based capture of targeted genomic regions which allows deep locus-specific sequencing and subsequently, high-resolution mapping of interactions between captured regions along with interactions between captured and non-captured regions. Generally, (1) A biotinylated RNA bait library is generated that specifically targets promoter-encompassing *Hin*dIII fragments. (2) Hi-C libraries are hybridized to the RNA baits to capture promoter-associated di-tags. (3) Hybridized libraries are sequenced [87]. This method has been applied to map 110 putative target genes to 33 breast cancer risk loci, uncovering long-range chromatin interactions. Such applications highlight CHi-C's utility in linking genetic variants at non-coding regions to their potential gene targets [88].

#### 2.5.2 ChIA-PET

ChIA-PET combines chromatin immunoprecipitation (ChIP) with 3 C to produce a protein factor-specific map of long-range contacts. In ChIA-PET, (1) Long-range chromatin interactions are crosslinked. (2) DNA-protein complexes are sonicated and enriched by chromatin immunoprecipitation (ChIP). (3) Tethered DNA fragments in each of the chromatin complexes are ligated with proximal DNA linkers. (4) Paired-End Tags (PETs) are extracted for sequencing and mapped to reference genomes. ChIA-PET results revealed close spatial proximity of distant chromosomal regions and protein factors [89].

#### 2.5.3 HiChIP

HiChIP further improves protein factor-directed identification of chromatin interactions through combining in situ Hi-C procedure in the nucleus and transposase-mediated on-bead library construction. Briefly, (1) Long-range DNA contacts are first fixed in situ in the nucleus to improve DNA contact capture efficiency. (2) Nuclei are lysed and sonicated. (3) ChIP is performed on the contact library, directly capturing elements of interactions including a protein of interest. (4) Paired-end sequencing is carried out to identify the interaction of the protein of interest with distant regions of the genome [90]. HiChIP dataset can be analyzed with the HiC-Pro pipeline which filters out unique paired-end tags. Using Hichipper chromatin loops coordinates, interaction frequency and statistical significance can be obtained [28], [90], [91]. Later, Ocean-C [92] and HiCoP [93] were developed to enrich the chromatin interactions of protein-free DNA regions.

### 2.6 Multimodal approaches to chromatin architecture

#### 2.6.1 Ocean-C

Open chromatin enrichment and network Hi-C (Ocean-C) incorporates Hi-C and FAIRE-seq to detect cis regulatory elements (CRE)-enriched open chromatin interactions. Ocean-C has an additional step of phenol-chloroform extraction after the biotinylation and sonication steps of Hi-C which specifically enriches re-ligation of open chromatin regions of nucleosome-free DNA fragments. Ocean-C enables identification of hubs of open chromatin interactions (HOCIs) in human cells. HOCIs are mainly active promoters and enhancers bound by a cluster of DNA-binding proteins, forming interaction networks strongly associated with gene expression, super-enhancers, and broad promoter domains [92].

#### 2.6.2 HiCoP

HiCoP couples Hi-C with CoP-seq to quantify the proximity of the accessible chromatin. Column Purified chromatin (CoP-seq) is a new method of detecting open chromatin that takes advantage of DNA purification columns to adsorb naked DNA fragments [93]. In HiCoP (1) DNA is crosslinked with formaldehyde and sonicated. (2) chromatin is purified on a PCR purification column. Thus, in HiCoP, after sonication Hi-C products are passed through a PCR column for purification followed by library construction and sequencing. While CoP-seq captures a subset of accessible chromatin located at active promoter regions, HiCoP detects more enhancer-associated active promoters. Interestingly, the unique HiCoP peaks are wide and weak, usually located at enhancer regions. HiC-Pro can be used to map HiCoP reads to human genome. Interaction loops can be detected using FitHiC and annotated with UCSC functional DNA elements [58], [59], [93].

#### 2.6.3 HiCAR

A rather recent advancement in observing 3D genome organization investigates genome-wide chromatin accessibility and CRE-anchored chromatin interactions. Hi-C on accessible regulatory DNA (HiCAR) leverages principles of in situ Hi-C and Tn5-mediated open-chromatin transposition, without utilizing antibodies or capture probes to pull down CRE sequences [94]. HiCAR workflow is as follows: (1) Cells are crosslinked and tagmented with a DNA adaptor-ligated Tn5 transposase. The Tn5 adapter contains a mosaic end (ME) sequence for Tn5 recognition and a single-stranded flanking sequence that can be ligated to the genomic DNA. (2) Digestion by restriction enzyme, in this case CviQI. (3) In situ proximity ligation to integrate the Tn5 adapter to spatially proximal genomic DNA. (4) Reversing crosslinks and DNA purification. (5) Digestion of the purified DNA with *Nla*III restriction enzyme. (6) Circularizing DNA by intramolecular ligation. (7) Library preparation and sequencing. HiCAR is used to identify functional CRE interactions that regulate gene expression. When combined with ChIP-seq data, HiCAR captures both active and poised CREs, demonstrating both active and poised interactions are enriched in compartment A, while depleted in B [94].

#### 2.6.4 Trac-looping

Trac-looping is a non-ligation technique that integrates chromatin accessibility and whole genome chromatin interactions [95]. Tn5-transposase is used to trace accessible chromatin regions and a ‘bivalent ME linker’ was specifically designed to capture genomic chromatin interactions of open chromatin regions. The bivalent ME linker is a mosaic end sequence containing a pair of MEs and a 30-bp oligonucleotide spacer that favors the formation of a Tn5 tetramer complex to capture two pieces of spatially proximal open-chromatin sequence, while the short spacer does not have the flexibility to allow intramolecular dimerization of the transposase–ME complex. The tetramer can be inserted to the genome in cis and in trans, thereby, crosslinking the interacting chromatin regions by the bivalent ME linkers and identifying chromatin interactions. Trac-looping can be used to capture accessible regions, chromatin secondary structure that includes short-range interactions spanning several nucleosomes, promoter and enhancer-associated chromatin interaction, as well as chromatin compartmentalization and TADs [95].

### 2.7 Single cell methods

Single cell Hi-C allows for detection of thousands of simultaneous chromatin contacts and mapping of 3D chromosome features in a single cell. However, dynamic nature of 3D genome structure poses debates on how genomic and transcriptomic status of a cell correlate and if transcriptome changes precede or follow the 3D chromatin structural modifications [96]. Multimodal scHi-C techniques such as single-nucleus methyl-3C sequencing (sn-m3C-seq) and scMethyl Hi-C were developed to improve 3D genome structure at single cell level. Both techniques are ligation-based and enable simultaneous profiling of chromatin conformation and methylation. As cytosine methylation (mC) remains unchanged during 3 C or Hi-C procedures, it allows combination of chromatin 3D capture with bisulfite sequencing [97].

#### 2.7.1 sn-m3C-seq

To perform single-nucleus methyl-3C sequencing (sn-m3C-seq), (1) Restriction enzyme digestion and ligation are performed on fixed nuclei. (2) Ligated nuclei are then distributed into 384-well PCR plates using fluorescence-activated nuclei sorting (FANS). (3) Proteinase digestion and bisulfite conversion are performed. (4) The libraries are constructed and sequenced. sn-m3C-seq data demonstrated that the differential DNA methylation signatures are associated with specific chromatin features.

As read alignment poses challenges for both Hi-C/3C and DNA methylation, a mapping pipeline for m3C-seq data was developed named “two-step alignment with unmapped reads using read splitting for methyl-Hi-C” (TAURUS-MH) that showed higher accuracy, mappability and long-range cis-contacts compared to conventional pipelines. TAURUS-MH uses a hybrid of ungapped and read splitting alignments. Sequencing reads will first be mapped to an in silico bisulfite converted genome, using bowtie, and then the unmapped reads will be split into three segments to undergo ungapped alignment [16], [97].

#### 2.7.2 scMethyl Hi-C

Methyl Hi-C is a technique that combines common in situ Hi-C procedure with an additional bisulfite conversion step (whole-genome bisulfite sequencing; WGBS) just before library preparation and paired-end sequencing. (1) Proximity ligation is performed on cells. (2) Individual nuclei are sorted into 96-well plates to separately undergo bisulfite conversion in each nucleus. (3) Library is constructed and sequenced. ScMethyl Hi-C enables characterizing cluster-specific chromosome conformation in complex tissues, concurrent with distinct TAD compartments between clusters and differentially methylated regions (DMRs) [98].

#### 2.7.3 HiRES

Hi-C and RNA-seq employed simultaneously (HiRES) is a ligation-based single cell multimodal technique [96]. HiRES procedure is as follows: (1) An in situ reverse transcription step. (2) A Hi-C procedure. (3) Flow sorting single cells. (4) Pre-amplification of single cells by quasilinear amplification of multiple annealing and looping-based amplification cycles in a one-tube reaction. (5) Extraction of the cDNA reads in silico through an mRNA-specific tag introduced through reverse transcription. Given the concurrent processing of DNA and RNA in HiRES, sample loss and handling time can be minimized. Also, the process is time efficient as one can process thousands of single cells within a few days by utilizing the multiwell plates. In addition, the throughput of the assay can be improved with multiplex single-cell barcodes. HiRES makes capture of cell cycle dynamics possible and enables exploration of the functional relationship between the 3D genome features and transcriptome in a single cell [96].

#### 2.7.4 scCARE-seq

Single-cell chromatin architecture and mRNA expression sequencing (scCARE-seq) is another single cell multimodal omics method for simultaneous detection of chromatin architecture and transcriptome in an individual cell. In scCARE-seq, (1) reverse transcription, digestion and ligation are performed in intact nuclei. (2) Individual cells are flow sorted into separate wells, and lysed. (3) Tn5 transposase is added to each well. (4) Each cell library is pre-amplified and split into two halves for separate library amplification of DNA and RNA by their corresponding primer pairs. (6) Nuclei are diluted and sorted by FACS. scCARE-seq data suggest parallel occurrence of periodic changes in chromatin architecture, and in transcription during the cell cycle [99].

#### 2.7.5 LiMCA

LiMCA (Linking mRNA to Chromatin Architecture) is a recent multimodal sequencing-based assay that concurrently measures genome-wide DNA contacts and full-length mRNA transcripts within individual cells [100]. LiMCA preserves the sensitivity and performance of each modality by physically separating the nucleus and cytoplasm of the same cell. The procedure involves the following steps: (1) The separated cytoplasm is subjected to Smart-seq2 for transcriptome analysis, enabling the capture of full-length mRNA transcripts. (2) The nucleus is processed separately using the standard Hi-C procedure to map chromatin contacts. (3) To enhance chromatin contact detection in single cells, a modified high-coverage transposon-based (META) whole-genome amplification (WGA) method is applied to the nucleus. (4) The Hi-C and mRNA libraries are then sequenced and integrated to provide a comprehensive view of chromatin architecture linked to gene expression.

LiMCA revealed that genes with higher expression levels tend to be located in the nuclear interior. Additionally, results from LiMCA were integrated with single-cell assay for transposase-accessible chromatin using sequencing (scATAC-seq) data, termed METATAC, to incorporate chromatin accessibility information alongside chromatin conformation and gene expression data [100], [101].

## 3 Discussion

The mapping of 3D genome architecture has evolved significantly over the past two decades, leading to the development of a wide array of techniques. These can broadly be categorized into ligation-based and ligation-free methods, each with distinct advantages, limitations, and specific applications. Here we compare these two categories, outline their applications, and explore future directions for 3D genome mapping, particularly focusing on multimodal approaches that integrate chromatin architecture with other layers of genomic information at both bulk and single-cell levels (Table 1).

**Table 1:** Overview of methods for 3D genome mapping and their applications. This table provides a comprehensive summary of various techniques used to study the three-dimensional architecture of the genome. The methods are categorized by their approach (ligation-based, ligation-free, targeted, and multimodal), indicating whether they are used in bulk or single-cell contexts. The table also highlights whether each method is targeted or multimodal and describes any additional modalities integrated with 3D genome mapping, offering a brief overview of each method's core features and applications.

Method Name	Type of Method	Bulk/Single Cell	Targeted/Multimodal	Additional Modality	Brief Description	Reference
**3 C**	Ligation-based	Bulk	-	-	First method for capturing chromatin interactions, identifies one-to-one interactions between loci.	Ref. [7]
**4 C**	Ligation-based	Bulk	-	-	Allows for one-vs-all interaction profiling, expands on 3 C by capturing interactions with a single locus.	Ref. [8]
**5 C**	Ligation-based	Bulk	-	-	Provides higher throughput than 3 C/4 C, capturing many-to-many chromatin interactions.	Ref. [9]
**Hi-C**	Ligation-based	Bulk	-	-	Maps all possible chromatin interactions genome-wide, provides comprehensive 3D genome architecture.	Ref. [10]
**Micro-C**	Ligation-based	Bulk	-	-	Offers nucleosome-resolution chromatin interaction mapping, particularly for short-range contacts.	Ref. [66]
**scHi-C**	Ligation-based	Single Cell	-	-	Captures 3D genome organization at single-cell resolution, highlighting cell-to-cell variability.	Ref. [71]
**sci-HiC**	Ligation-based	Single Cell	-	-	Massively multiplexed single-cell Hi-C for high-throughput mapping of chromatin interactions.	Ref. [72]
**sc-DipC**	Ligation-based	Single Cell	-	-	Differentiates between chromosomal haplotypes, detecting more true contacts via phasing SNPs.	Ref. 75
**scNanoHiC**	Ligation-based	Single Cell	-	-	Uses long-read sequencing to profile high-order chromatin structures in individual cells.	Ref. [76]
**Droplet HiC**	Ligation-based	Single Cell	-	-	Combines in situ Hi-C with microfluidics to profile chromatin architecture in single cells.	Ref. [77]
**GAM**	Ligation-free	Bulk	-	-	Captures 3D chromatin proximities without ligation, suitable for multi-way interactions.	Ref. [79]
**Multiplex-GAM**	Ligation-free	Bulk	-	-	An affordable variant of GAM, allows mapping of more chromatin contacts genome-wide.	Ref. [81]
**SPRITE**	Ligation-free	Bulk	-	-	Detects higher-order interactions simultaneously between multiple DNA sites within the same nucleus.	Ref. [82]
**Single Chromosome Mapping**	Ligation-free	Single Cell	-	-	Visualizes chromatin domains and TADs within individual chromosomes using FISH.	Ref. [83]
**ChIA-Drop**	Ligation-free	Single Cell	-	-	Barcodes chromatin complexes inside droplets for mapping multiplex interactions with single-molecule precision.	Ref. [85]
**scSPRITE**	Ligation-free	Single Cell	-	-	Provides high-resolution contact maps at the single-cell level by capturing multi-way DNA interactions.	Ref. [86]
**Capture-HiC**	Targeted	Bulk	Targeted	-	Uses RNA baits to capture promoter-associated chromatin interactions with high resolution.	Ref. [87]
**ChIA-PET**	Targeted	Bulk	Targeted	-	Combines ChIP with 3 C to produce protein factor-specific chromatin interaction maps.	Ref. [89]
**HiChIP**	Targeted	Bulk	Targeted	-	Enhances protein factor-directed identification of chromatin interactions using in situ Hi-C.	Ref. [90]
**Ocean-C**	Multimodal	Bulk	Multimodal	Chromatin Accessibility	Enriches open chromatin interactions to identify hubs of chromatin interactions linked to gene regulation.	Ref. [92]
**HiCoP**	Multimodal	Bulk	Multimodal	Chromatin Accessibility	Detects accessible chromatin regions and their proximity using Hi-C coupled with CoP-seq.	Ref. [93]
**HiCAR**	Multimodal	Bulk	Multimodal	Chromatin Accessibility	Maps genome-wide chromatin accessibility and regulatory interactions without antibodies or capture probes.	Ref. [94]
**Trac-looping**	Multimodal	Bulk	Multimodal	Chromatin Accessibility	Integrates chromatin accessibility with genome-wide chromatin interactions using a bivalent linker.	Ref. [95]
**sn-m3C-seq**	Multimodal	Single Cell	Multimodal	DNA Methylation	Simultaneous profiling of 3D genome structure and DNA methylation at single-cell level.	Ref. [97]
**scMethyl-HiC**	Multimodal	Single Cell	Multimodal	DNA Methylation	Combines in situ Hi-C with bisulfite sequencing for simultaneous chromatin conformation and methylation profiling.	Ref. [98]
**HiRES**	Multimodal	Single Cell	Multimodal	Transcriptomics	Captures 3D genome structure and RNA expression simultaneously in single cells.	Ref. [96]
**scCARE-seq**	Multimodal	Single Cell	Multimodal	Transcriptomics	Simultaneous detection of chromatin architecture and transcriptome in individual cells.	Ref. [99]
**LiMCA**	Multimodal	Single Cell	Multimodal	Transcriptomics	Concurrently measures genome-wide DNA contacts and full-length mRNA transcripts in individual cells.	Ref. [100]

### 3.1 Ligation-based vs. ligation-free methods

Ligation-based methods, such as Hi-C and its derivatives, have been the cornerstone of 3D genome mapping. These methods rely on crosslinking chromatin, digestion with restriction enzymes, and ligation of proximally interacting DNA fragments to generate libraries that can be sequenced to reveal chromatin contacts. Ligation-based methods are particularly well-suited for comprehensive mapping of all chromatin interactions within a genome, making them ideal for studies aiming to uncover the overall 3D architecture of the genome, including the identification of TADs, compartments, and chromatin loops. Applications of ligation-based methods are extensive, including the reconstruction of whole genome contacts, scaffolding and assembly of genomes, haplotype phasing, centromere annotation, and the investigation of gene regulatory mechanisms. The scalability of these methods also allows them to be adapted for single-cell analyses, enabling the exploration of cell-to-cell variability in chromatin organization. However, ligation-based methods come with certain limitations. They are prone to biases introduced by restriction enzyme digestion, ligation efficiency, and sequence mappability, which can affect the accuracy of the resulting contact maps. The resolution of these methods is dependent on the frequency of restriction sites across the genome, which may limit the ability to resolve fine-scale chromatin interactions. Additionally, the workflows are often complex, involving multiple enzymatic steps that can introduce noise and artifacts, particularly in single-cell applications [7], [8], [9], [10], [66], [69], [72], [75], [76].

Ligation-free methods, such as GAM and SPRITE, address some of the limitations of ligation-based approaches by bypassing the need for enzymatic ligation of DNA fragments. These methods can capture multi-way interactions and provide insights into chromatin organization that may be difficult to obtain using ligation-based techniques. For instance, GAM and SPRITE excel in detecting higher-order interactions involving multiple genomic loci, offering a more nuanced view of nuclear architecture. These methods are particularly advantageous when studying complex chromatin interactions that involve multiple regions or when there is a need to avoid biases associated with enzymatic steps. Ligation-free methods are preferable for studies aiming to capture multi-way interactions or when the focus is on interactions involving multiple DNA sites within the same nucleus. They are also more suitable when there is a need to avoid the biases introduced by ligation, such as when investigating interactions in regions of the genome with low restriction enzyme site density or when studying chromatin associations with nuclear bodies and the nuclear periphery. Additionally, these methods can be particularly valuable in single-cell applications where they enable the capture of cell-to-cell variability in complex chromatin interactions. However, ligation-free methods may present challenges in terms of resolution. While they excel in capturing higher-order interactions, they may lack the resolution offered by high-coverage ligation-based methods like Hi-C. The workflows for these methods, particularly at the single-cell level, can also be technically challenging. Moreover, the data generated by ligation-free methods can be more complex to interpret, especially when integrating it with existing Hi-C datasets [3], [79], [81], [83], [86].

When choosing between ligation-based and ligation-free methods, we should consider the specific goals of the study. For comprehensive genome-wide mapping of chromatin interactions, particularly if the focus is on TADs, loops, and overall genome architecture, ligation-based methods like Hi-C and its variants are often the preferred choice. These methods provide high-resolution maps that are essential for understanding the spatial organization of the genome and its impact on gene regulation. In contrast, if the study aims to investigate complex multi-way interactions, such as those involving multiple genomic regions or interactions with nuclear bodies, ligation-free methods like GAM and SPRITE are more suitable. These methods are particularly valuable for investigating interactions that may not be easily detected by ligation-based approaches due to the limitations of restriction enzyme digestion and ligation steps.

## 4 Future directions and multimodal approaches

Recent developments, such as HiCAR and HiChIP, have combined chromatin interaction mapping with chromatin accessibility and protein-DNA interaction data, respectively. These methods allow us to link chromatin architecture with regulatory element activity and gene expression, shedding light on the functional consequences of 3D genome organization [90], [94]. Bulk multimodal approaches are particularly useful in studies aiming to understand how chromatin architecture influences gene regulation across different cell types and conditions.

At the single-cell level, methods like LiMCA and scCARE-seq have emerged as powerful tools to simultaneously capture chromatin architecture and transcriptomic data. These techniques preserve the spatial and transcriptional context within individual cells, enabling us to explore the interplay between chromatin structure and gene expression on a cell-by-cell basis [99], [100]. Single-cell multimodal approaches are essential for studying cellular heterogeneity and understanding how different layers of genomic information interact within individual cells.

The future of 3D genome mapping lies in the continued development and refinement of multimodal approaches. Emerging technologies that combine chromatin conformation with epigenomic and transcriptomic profiling at single-cell resolution will enable unprecedented insights into the regulatory dynamics underlying development, disease, and cellular heterogeneity. Additionally, improvements in computational tools and machine learning algorithms will be essential for integrating and interpreting these complex datasets, ultimately driving new discoveries in genome biology.

## CRediT authorship contribution statement

**Elias Orouji:** Writing – review & editing, Writing – original draft, Visualization, Supervision, Resources, Project administration, Methodology, Investigation, Funding acquisition, Data curation, Conceptualization. **Ghazaleh Tavallaee:** Writing – review & editing, Writing – original draft, Visualization, Resources, Project administration, Methodology, Investigation, Conceptualization.

## Declaration of Competing Interest

The authors declare that they have no known competing financial interests or personal relationships that could have appeared to influence the work reported in this paper.
